# Looking Back to Move Forward: A Narrative Review of Indigenous Health Intervention Research by the University Departments of Rural Health Against a Contemporary National Framework

**DOI:** 10.3390/ijerph23050600

**Published:** 2026-05-01

**Authors:** Katrina Fyfe, Samantha Bay, Emma V. Taylor, Ha Hoang, Lisa Hall, Annette McVicar, Emma Walke, Carolyn Lethborg, Bahram Sangelaji, Sandra C. Thompson

**Affiliations:** 1 Western Australian Centre for Rural Health (WACRH), University of Western Australia, P.O. Box 109, Geraldton, WA 6531, Australia; samantha.bay@uwa.edu.au (S.B.); emma.taylor@uwa.edu.au (E.V.T.); 2 Centre for Rural Health, School of Health Sciences, University of Tasmania, Locked Bag 1322, Launceston, TAS 7250, Australia; thi.hoang@utas.edu.au; 3 Rural Health Bendigo, Monash University, 26 Mercy Street, Bendigo, VIC 3552, Australia; lisa.hall@monash.edu.au; 4 Western Australian Centre for Rural Health (WACRH), University of Western Australia, P.O. Box 62, Karratha, WA 6714, Australia; 5 University Centre for Rural Health, University of Sydney, 61 Uralba St., Lismore, NSW 2480, Australia; emma.walke@sydney.edu.au; 6 St. Vincent’s Hospital, 41 Victoria Parade, Fitzroy, VIC 3065, Australia; carolyn.lethborg@svha.org.au; 7 Southern Queensland Rural Health, University of Queensland, Boyce Ave., Cranley, QLD 4350, Australia; b.sangelaji@uq.edu.au

**Keywords:** indigenous, policy, health, interventional research, rural

## Abstract

**Highlights:**

**Public health relevance—How does this work relate to a public health issue?**

- Assesses the alignment of historical rural Indigenous health intervention research with the National Aboriginal and Torres Strait Islander Health Plan 2021–2031, a central national public health policy framework.
- The Framework is important in addressing persistent public health inequities experienced by Indigenous peoples in rural and remote Australia, including systemic racism, workforce capacity, and access to culturally safe care.

**Public health significance—Why is this work of significance to public health?**

- Provides a systematic, national appraisal of whether historical Indigenous-focused rural health research has been aligned with contemporary Indigenous-led public health priorities and reform agendas.
- Identifies there have been gaps in intervention research targeting social and emotional wellbeing, mental health, and suicide prevention—areas with substantial impact on Indigenous health outcomes.

**Public health implications—What are the key implications and messages for practitioners, policy makers, and/or researchers in public health?**

- The Australian University Departments of Rural Health (UDRHs) have a long history of engaging in Indigenous health research, including efforts at interventions undertaken in partnership with rural Indigenous communities.
- There are opportunities to strengthen closing the gap through meeting contemporary expectations for transparency, shared data access, and alignment with national Indigenous health priorities to maximise public health impact.

**Abstract:**

The Australian University Departments of Rural Health (UDRHs) promote the health and wellbeing of people in rural and remote Australia through health education, research, and advocacy. This narrative review evaluated the extent to which Indigenous health intervention research conducted by UDRHs over a 12-year period (2010–2021) aligned with the Principles and Priorities of the National Aboriginal and Torres Strait Islander Health Plan 2021–2031. The purpose was to reflect on past UDRH research contributions to identify existing strengths and areas for improvement in line with current policy. Thirty-three relevant UDRH publications were identified from a broader database of UDRH research outputs. Each paper was independently coded by at least two authors as demonstrating “yes”, “partial”, or “not evident” alignment with the twelve priorities of the Health Plan. UDRH intervention research demonstrated strengths in genuine shared decision making and partnerships with Indigenous communities, workforce development, health promotion, and identifying and addressing racism. However, gaps were evident in research addressing social and emotional wellbeing, mental health and suicide prevention, promotion of healthy environments, sustainability and preparedness, and transparency regarding shared access to data and information. UDRHs play a key role in building research capacity among staff and communities in rural settings and often maintain long-standing, respectful relationships with local Indigenous communities. While UDRH research aligns with many domains of the national Health Plan, future efforts should prioritise social and emotional wellbeing and mental health. Improved reporting of shared data access represents an immediate opportunity for enhancement.

## 1. Introduction

Aboriginal and Torres Strait Islander peoples (hereafter, respectfully referred to as Indigenous), the original custodians of Australia, continue to experience significant health inequities and broader social disparities, stemming from the enduring legacy of colonisation and systemic disadvantage. Factors contributing to the disparity in burden of disease between Indigenous and non-Indigenous Australians are estimated at 35% from social determinants of health (e.g., employment, income, education, housing) and 30% from selected health risk factors such as smoking [1]. The remaining 35% is “unexplained”, and the Australian Institute of Health and Welfare (AIHW) suggests that these unexplained factors may include “access to affordable and culturally appropriate health care services, connection to Country and language, and effects of structural disadvantage and racism” [1]. The 2024 Aboriginal and Torres Strait Islander Health Performance Framework–Report states that in 2018, Indigenous Australians experienced 2.3 times the burden of disease of non-Indigenous Australians [1].

For close to two decades, successive Australian governments have worked to overcome Indigenous Australians’ disadvantage and, recently, to work more closely with Indigenous leaders in developing policy and programmes. The current National Aboriginal and Torres Strait Islander Health Plan was published in 2021 [2] and built on successive and existing policies and the National Aboriginal and Torres Strait Islander Health Plan 2013–2023 [3]. These ten-year Health Plans are aligned with the Coalition of Aboriginal and Torres Strait Islander Organisations and Council of Australian Governments (COAG) and the National Reform Agreement, known as “Closing the Gap” [4] in Indigenous disadvantage. The Closing the Gap campaign started in 2005 after the Australian Aboriginal and Torres Strait Islander Justice Commissioner released the Social Justice Report 2005 [5]. This report called for Federal, State, and Territory governments in Australia to commit to achieving health and life expectancy equality within 25 years [5]. The campaign was formalised by the National Reform Agreement in 2009. The most recent Closing the Gap Agreement has four Priority Reforms: formal partnerships and shared decision making; building the community-controlled sector; transforming government organisations; and shared access to data and information at a regional level [6]. The Priority Reforms were directly informed by Indigenous people [6] and have clear connections to the National Aboriginal and Torres Strait Islander Health Plan Framework 2021–2031 [2] (hereafter referred to as the Framework).

**Figure 1:**
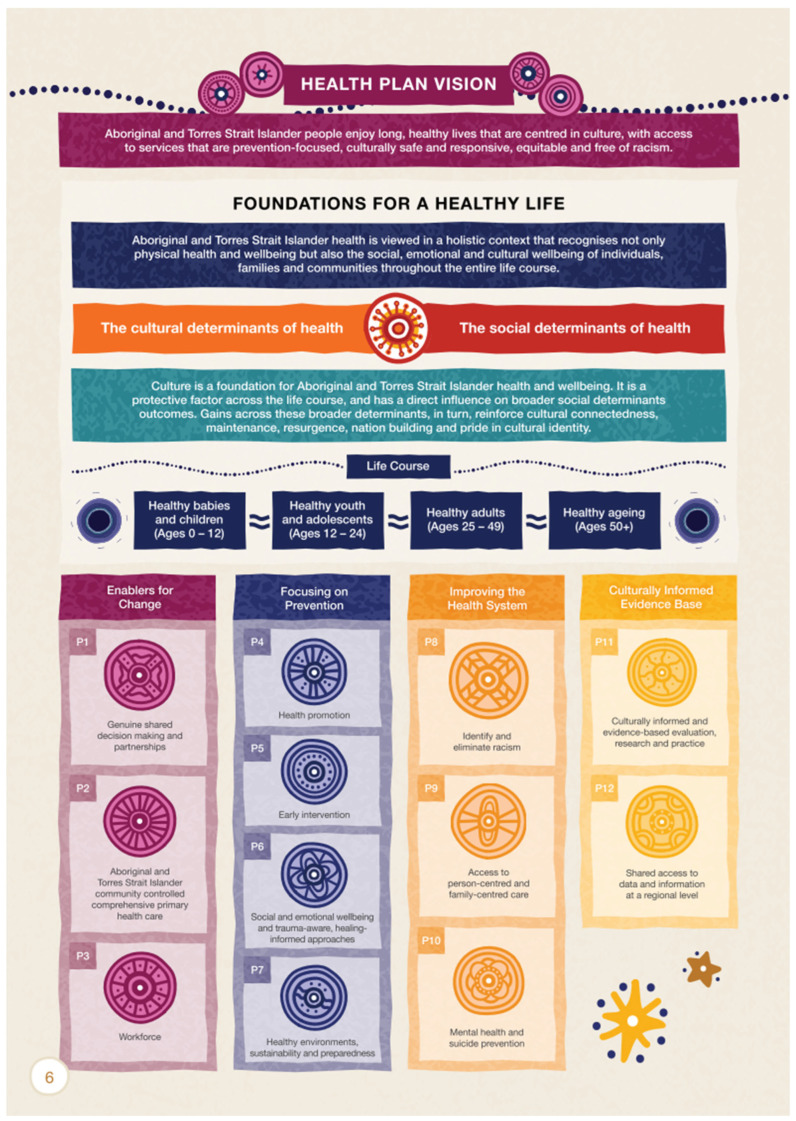
Health Plan Vision from the National Aboriginal and Torres Strait Islander Health Plan Framework 2021–2031 [2]. Reprinted with permission from the Australian Government’s National Aboriginal and Torres Strait Islander Health Plan 2021–2031 [2]. Artwork by Gilimbaa artist Tarni O’Shea (South Sea Islands/Butchulla).

The Framework consists of four overarching Principles and twelve Priorities, all interlinked with the concepts of a “Health plan vision” and “Foundations for a healthy life” across the life course. The key influences of “The cultural determinants of health” and “The social determinants of health” sit within the “Foundations for a Healthy Life”. The importance of Indigenous culture is woven throughout the Framework and appears prominently at every level. This document is more evolved than those that preceded it; its development the result of a genuine partnership between the Health Plan Working Group and Implementation Plan Advisory Group comprising Indigenous health experts and governments [2]. As well as the Framework being underpinned by the Closing the Gap Agreement, it also links with other key Commonwealth health policies [2]. Figure 1 shows the Health Plan Vision (page 6 from the Framework), which summarises the Principles and Priorities for Indigenous health [2].

**Figure 2:**
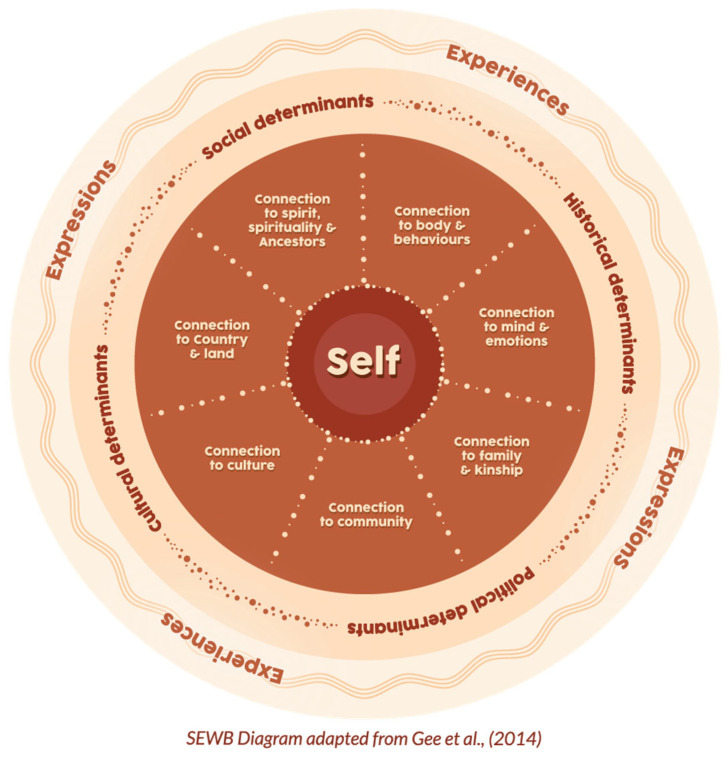
SEWB diagram adapted from Gee et al. (2014) as presented in Dudgeon et al. (2025) [7]. Reproduced with permission from Ref. [10]. 2014, Gee et al.

Within the Framework, recognition of the concept of Indigenous social and emotional wellbeing (SEWB) is formalised (via Priority 6 and woven throughout the document) [2]. SEWB is a relatively new concept that describes the Indigenous meaning of health as different from the Western view of health and wellbeing [7]. The concept was first recognised in 1970s Australia by the then peak body, the National Aboriginal and Islander Health Organisation (NAIHO), now the National Aboriginal Community Controlled Health Organisation (NACCHO) [7]. The National Aboriginal Health Strategy (1989) [8] progressed the idea, and this was followed by the development of nine Principles to guide understanding of Indigenous health and wellbeing in the Ways Forward Report (1995) [7,9]. A more refined understanding was developed in 2013 by Gee et al. (2014) [10] in consultation with Indigenous Australians [7,10]. Dudgeon et al. (2025) [7], in their narrative review of SEWB, present an adaptation of Gee et al.’s (2014) [10] conceptual model of SEWB (Figure 2).

SEWB is a complex vision of health that recognises that Indigenous “physical, mental, and spiritual health is strongly related to historical and cultural factors of connections to family, community, culture, Country, spirituality, and ancestors. In many ways, and for many Aboriginal and Torres Strait Islander peoples, physical, mental, and spiritual health are inextricably linked to having balanced, harmonious connections to kinship and culture and to the total wellbeing of their community and Country” [7]. Importantly, the nine principles, the SEWB concept, and the Framework recognise that there is a direct linkage between Indigenous mental wellbeing and the long-term impact of experiences of trauma and loss, particularly linked to European colonisation of Australia [2,7,10].

Longstanding government policies to build the health workforce through investment in rural academic centres have funded Rural Health Multidisciplinary Training across Australia. The University Departments of Rural Health (UDRHs) have been working in the rural and remote space, and numbers have steadily increased from two UDRHs in 1996 to 19 in 2023 [11]. The main roles of the UDRHs are to provide rural and remote training opportunities for nursing and allied health students, support the local health workforce with professional development, and to conduct high-quality, locally relevant rural health research. In Lyle and Greenhills’ (2018) [12] review of the contributions of the UDRHs, they detail “two decades of capacity building in rural health education, training and research” and concluded that “community engagement and accountability to region are hallmarks of the program” [12].

This study evaluates how intervention research in Indigenous health undertaken over a 12-year period by or in conjunction with UDRHs aligns with the current Framework’s Principles and Priorities [2]. It reflects on UDRH research contributions and gaps, and identifies potential areas for improvement and future research. As this paper aims to provide direction for future research, we used the latest version of the national Framework in our assessment. The current iteration of the Framework represents an evolution of the policy frameworks over time. The notable change is that the current Framework has been developed in consultation with Indigenous people.

## 2. Materials and Methods

### 2.1. Study Selection and Data Collection

The Australian Rural Health and Education Network (ARHEN) is the national association advocating for the 19 UDRHs, supporting collaboration to improve the health and wellbeing of rural and remote communities through education, research, and service development [11]. In 2019, ARHEN established an EndNote 21 (Clarivate, Philadelphia, PA, USA) database of published peer-reviewed research by UDRH contributors. Collaborators from UDRHs identified and analysed 493 papers about Indigenous health published between 2010 and 2021 to provide a broad overview of the nature of research across the 12-year period [13]. The team of researchers worked to review UDRH papers with a focus on Indigenous health, culture, and education, using a framework adapted from Sanson-Fisher and colleagues [14]. Research publications were grouped into the following subcategories: descriptive, intervention, and measurement. These are subclassified into broad descriptive categories: health services research; epidemiology; Indigenous culture and wellbeing; workforce issues; and other (to include clinical treatments).

As part of capacity building and enabling Indigenous input, Indigenous researchers from the UDRH—Aboriginal and Torres Strait Islander Staff Alliance were invited to participate in the Framework assessment. Those who self-nominated contributed to all aspects of the study, including the evaluation of papers against the Framework and writing the article. The preferred Aboriginal affiliation of each of these researchers has been noted with their agreement.

The 36 intervention research articles reported as part of the collection of papers examining UDRH Indigenous health research in Thompson et al.’s work [13] were subject to deeper interrogation in this study.

A subgroup of the research team (K.F., S.B., E.W., A.M., and S.C.T.) read the full text of these papers and extracted information in a custom table to capture key aspects of each. This included: the focus of the study; the type of study; the nature of the intervention; and other key information, tools employed in the delivery of the intervention, and barriers and enablers to success. The findings have been published elsewhere [15].

### 2.2. Ethics Approval

This study did not require ethics approval as it is a narrative review of published research papers.

### 2.3. Inclusion Criteria

Only articles describing completed Indigenous interventional research papers published with a UDRH author that appeared in the ARHEN EndNote 21 library [13] were included in this narrative review.

### 2.4. Exclusion Criteria

Of the 36 intervention papers, three were study protocol papers [16,17,18]. As the protocols described proposed interventions rather than completed interventions, they were excluded, leaving 33 articles for assessment against the Framework.

### 2.5. Mapping and Coding

Each of the 33 papers was independently examined by two authors (K.F. and S.B.) and mapped to the four Principles and the twelve Priorities of the Framework. The Framework [2] was regularly consulted to ensure accurate coding, and the two authors resolved discrepancies through discussion and consensus. Where discrepancies were unresolved, a third author (SCT) was consulted. In addition to summarising the data mapping, Table 1 lists all Priorities under their respective Principles, and Figure 1 gives a visual illustration of the same.

The 33 articles were assessed and coded as “yes”, “partially” or “not evident” for each Priority in the Framework. Articles were coded as “yes” if they described processes that matched the Priority description; “partially” if the article described some elements of the Priority; and “not evident” if there was no evidence or description of the study addressing the Priority.

For example, for Schoen et al. (2016) [19], Priority 2—Aboriginal and Torres Strait Islander community-controlled comprehensive primary health care, was coded as “yes” as it was stated in the article that “Study sites were 15 rural and remote towns in the Midwest and Pilbara regions of Western Australia. Fifteen workshops were delivered in hospitals, five in Aboriginal Community Controlled Health Organisations (ACCHOs), five at a rural health centre, two at aged care centres, and one each at a rural university centre, a remote nursing post and rural general practice” [19].

### 2.6. Data Analysis

After coding, a table was created to consolidate the final mapping with short descriptions of each intervention and the sample size of the intervention groups.

The data were entered into Microsoft Excel 365 (Microsoft^®^ Corp., Redmond, WA, USA) for further analysis. Counts of how many Priorities each article met were collated, along with counts of how many articles addressed all Priorities within each Principle. Additionally, the full-text articles were saved in an EndNote 21 library and tagged into Priority groups. Notations were made directly into the Portable Document Format (PDF) of each article within EndNote 21 by two authors (S.B. & K.F.) to highlight how each one met the specific Priority. A third author (S.C.T.) verified these notations.

## 3. Results

Mapping of 33 intervention articles against the Framework showed varying degrees of alignment with the Framework’s Principles and Priorities. Table 1 shows the number of articles meeting each Priority (P1–12) under the overarching Principle from the Framework. Supplementary Table S1: Summary of all papers and mapping to the National Aboriginal and Torres Strait Islander Health Plan 2021–2031, summarises the nature of interventions and assessments of the studies against the Framework.

Improving the health system by identifying and eliminating racism (Priority 8) was consistently featured across all intervention research, along with other Priorities represented in over half of the papers: genuine shared decision making and partnerships (Priority 1), health promotion (Priority 4), and culturally informed and evidence-based evaluation, research and practice (Priority 11). A small number of Priorities were under-represented in the reviewed articles. Notably, mental health and suicide prevention (Priority 10), healthy environments, sustainability and preparedness (Priority 7), and shared access to data and information at a regional level (Priority 12) were under-represented in UDRH publications.

### 3.1. Principle 1: Enablers for Change

The first Principle overarches Priorities 1, 2 and 3. Over half of all articles addressed Priority 1—genuine shared decision making and partnerships (*n* = 22; 67%) [20,21,22,23,24,25,26,27,28,29,30,31,32,33,34,35,36,37,38,39,40,41] and Priority 3—workforce (*n* = 19; 58%) [19,21,22,23,26,27,29,30,31,32,34,35,36,37,39,41,42,43,44]. Comparatively, Priority 2—Aboriginal and Torres Strait Islander community-controlled comprehensive primary health care, was the least addressed Priority under Principle 1 (*n* = 13; 39%) [19,20,26,27,30,31,35,36,37,41,42,45,46].

Some UDRH contributions demonstrated alignment with all three Priorities of the first Principle. For example, Chapple et al. (2016) [26] showed all priorities of the enablers for change Principle in their exploration of the Living Well Smoke Free training intervention, focusing on smoking cessation. However, while interviews reflected that the approach was culturally appropriate, they noted that giving up smoking was not a priority for their participants due to their “complex lives”, including having past and existing trauma such as being a part of the Stolen Generation, experiencing domestic violence, grief and loss. Khalil (2019) [30] also demonstrated all three priorities that enabled change in the development and implementation of a medication safety programme, undertaking the project in an ACCHO and working with Indigenous Health Practitioners, to build capacity in these workers and develop a culturally appropriate and useful program. Reeve et al. (2014) [36] evaluated an ear health program that also aligned with all Priorities of the first Principle; the program showed an increase in children being screened (from 148 to 710 within 18 months) and a substantial reduction in median waiting time for an Ear, Nose and Throat (ENT) review using telehealth (from 141 days to 22 days). The successful outcomes were achieved by developing a new electronic referral template that was integrated within a telehealth service, through working in partnership with the local cultural health services, and through building capacity in ear health among Australian Indigenous health workers.

UDRH contributors demonstrated understanding of the importance of consultation with Indigenous communities and capacity building, and utilised these enablers of change to improve Indigenous healthcare services. For example, Rae et al.’s (2014) [34] article detailed local pregnant Indigenous women being engaged in research to reduce renal disease and improve birth outcomes through interactions with the Artshealth programme, developed in consultation with local Elders in 2007 and reported by the authors as the largest longitudinal study of its kind internationally. Art sessions were directed by Indigenous artists in a strength-based approach, alongside conversations between pregnant Indigenous women and health professionals to build trust and health-related knowledge. The researchers noted that as they “developed increasingly strong relationships with the women…we have been rewarded by our participants suggesting health programs” [34]. Durey et al. (2016) [27] described their “unique strategy of community engagement…to design culturally-responsive healthcare…”. Using a genuine shared decision making and partnership approach was also evident in Kong et al. (2021) [31] who employed a Participatory Action Research approach to develop and pilot an Indigenous culturally appropriate version of the Midwifery Initiated Oral Health (MIOH) model of care.

### 3.2. Principle 2: Focusing on Prevention

The second Principle overarches Priorities 4, 5, 6, and 7. Twenty-one articles aligned with Priority 4—health promotion [19,21,22,24,25,26,27,28,29,30,31,32,34,35,36,37,41,43,45,47,48]—fifteen addressed Priority 5—early intervention [19,24,28,29,31,32,34,35,36,42,43,45,46,47,49]—and four articles addressed Priority 6—social and emotional wellbeing and trauma-aware, healing-informed approaches [22,35,38,45]. One article partially addressed Priority 7—healthy environments, sustainability, and preparedness—by advocating for a return of water fluoridation in a specific Indigenous community so as to reduce dental caries after this government service had ceased in mid-2011 [47].

Overall, studies that addressed Principle 2 covered diverse topics including interventions aimed at improving dental, ear and renal health in Indigenous children [34,36,47,49], consumer behaviour in food and drink purchases in remote Indigenous communities [25], smoking cessation programmes [26,32], secondary prophylactic approaches for management of rheumatic heart disease [43,46] and point of care testing for diabetes and chlamydia [24,37]. The standout article that focused on SEWB (and Priority 6) was Bennett-Levy et al. (2021) [22], who state that they believed the WellMob site they developed to be the first “dedicated one-stop-shop digital SEWB website for Indigenous peoples anywhere in the world”.

### 3.3. Principle 3: Improving the Health System

Principle 3 overarched Priorities 8, 9, and 10. The most addressed Priority was Priority 8—Identifying and eliminating racism. Thackrah et al. (2013, 2013, 2015, 2015) [38,39,40,48] produced a series of papers describing the “uncomfortable truths” of “receptivity and resistance to Aboriginal content in midwifery education”, and how including Indigenous content, and clinical placements with Indigenous women in remote areas of Western Australia were able to promote culturally respectful practice.

While none of the UDRH studies explicitly aimed at overcoming racism, interventions were considered to indirectly identify and eliminate systemic racism. All papers acknowledged the importance of the inclusion of Indigenous Australians in developing health resources and approaches to care. Studies highlighted disparities in Indigenous health outcomes and noted that reduced institutional racism was associated with improved care [25,27]. All except one article acknowledged the disparities in health outcomes between Indigenous and non-Indigenous people as a rationale for targeting interventions that aimed to provide better care in Indigenous health [19,20,21,23,24,25,26,27,28,29,30,31,32,33,34,35,36,37,38,39,40,41,42,43,44,45,46,47,48,49,50,51]. Although one article did not clearly articulate disparities in Indigenous health, the processes described in engaging Indigenous communities influenced the Australian government’s digital mental health programme to ensure it aligned with a “culturally-relevant Indigenous framework (and resources) of digital social and emotional wellbeing…” [22], thus contributing to eliminating systemic racism.

Access to person-centred and family-centred care (Priority 9) was represented in the articles, with eight articles mapped as “yes” [22,32,34,35,36,41,48,50] and six as “partially” [25,26,29,38,42,45]. For example, Carey et al. (2016) [50] included two case studies in their paper describing a palliative care respite service that demonstrated person-centred care. One case involved a client who took care of the garden and acted as an interpreter for other clients when he was at the respite centre. This resulted in him feeling that he had a genuine role in the house, and his emergency department attendances (reported as chiefly for pain management and to alleviate his isolation) dropped from 35 to 5 times over twelve months, his feelings of isolation were reduced and symptom management improved [50].

Only three papers specifically addressed Priority 10—mental health and suicide prevention [21,22,41]. The three papers described the barriers and enablers to uptake of by health professionals of an e-health intervention that highlighted the need to match the type of resources to work roles [21], a six year project that trained Indigenous health professionals in digital mental health resulting in development of the Wellmob Website that remains active today (https://wellmob.org.au/ accessed on 13 March 2025) [22], and piloting an Indigenous male health module, which flagged promotion and access to the module as being primary to its success [41]. Development of the Wellmob Website was arguably the most successful of these projects, attributed to the “community-based participatory research” methods that were used, and that “the guidance from the local Indigenous community partners went on to influence the national government’s digital mental health agenda” from digital mental health to the “culturally-relevant Indigenous framework of digital social and emotional wellbeing” [22]. This project also directly recognised the important link between SEWB and mental health, which ties together many sections of the paper [22].

### 3.4. Principle 4: Culturally Informed Evidence Base

Principle 4 overarches Priorities 11 and 12. Nineteen articles [20,21,22,23,25,26,27,29,30,31,32,33,34,35,37,38,39,40,41] aligned with Priority 11—culturally informed and evidence-based evaluation, research and practice—with the desired outcome of “being future-focused, and Aboriginal and Torres Strait Islander led research and evaluation. The experiences, knowledge and expertise of Aboriginal and Torres Strait Islander people is embedded across policy and program development” [2]. The articles described using culturally informed and evidence-based approaches to evaluation, research and/or practice, which were also associated with co-design practices in Principle 1—enablers for change. For example, Cairns et al. (2022) [25] used an action research approach to co-design a community rehabilitation and lifestyle service and discovered that this service required three key elements for culturally responsive care. Prout et al. (2014) [33] reported on the experiences of health students attending Country Week, particularly on their preparation for working in a rural/remote area with Indigenous people. The students were introduced to local health practitioners and Indigenous people who talked about their life experiences and Indigenous history. Students were encouraged to maintain a reflective journal of their time at Country Week, with the trip proving to be a powerful, transformative learning experience, which hopefully translated into culturally informed practice in the future [33].

Only six articles described addressing Priority 12—shared access to data and information at a regional level [22,27,30,34,35,41]—and how they achieved local Indigenous engagement and addressed local priorities. More than one approach was used; for example, Bennett-Levy et al. (2021) [22] developed digital resources for Indigenous health professionals via a community-based participatory approach and achieved data and information sharing through the Wellmob Website [52]. Reeve et al. (2015) [35] set up a formal partnership between Fitzroy Hospital, community health services and the community-controlled health service.

## 4. Discussion

This analysis evaluates how intervention research in Indigenous health undertaken by UDRHs aligns with the current national Framework’s Principles and Priorities. Our assessment highlighted areas of research strengths in genuine shared decision making and partnerships, involving Aboriginal and Torres Strait Islander community-controlled comprehensive primary health care, workforce interventions, health promotion, identifying and eliminating racism, and using culturally informed and evidence-based evaluation research and practice. UDRH roles in research projects could be strengthened in planning, implementing, and evaluating interventions; areas for attention are improving mental health, SEWB, and suicide prevention [53]. Greater efforts for transparency in the sharing of data and information at a regional level, and reporting on this, need to be consistent with Indigenous Data Sovereignty [54]. This sharing of information requires the commitment of all stakeholders to come together to learn from projects and research being developed and undertaken to maximise the benefits of research, knowledge transfer, and translation. Improved transparency of reporting in publications can be acted upon immediately by UDRHs.

Most studies showed some alignment with all four Principles of the Framework, with most aligning or partially aligning with at least one Priority in each Principle. UDRH research published in the previous 12 years was relevant to the priorities of Indigenous communities in the current Health Plan 2021–2031. This is unsurprising given that the current Framework evolved from previous national Indigenous Health Plans, UDRHs have a history of partnerships and engagement with Indigenous communities, and UDRHs employ Indigenous staff and researchers who are involved in Indigenous health projects. It was evident that UDRH research has contributed to improving the health system, particularly through efforts in reducing systemic racism, which was evident in all 33 publications. Many studies demonstrated genuine shared decision making and partnerships [20,21,22,23,24,25,26,27,28,29,30,31,32,33,34,35,36,37,38,39,40,41], with ACCHOs’ care [19,20,26,27,30,31,35,36,37,41,42,45,46], and capacity building of the workforce in Indigenous health services [19,21,22,23,26,27,29,30,31,32,34,35,36,37,39,41,42,43,44]. Many projects focused on prevention through health promotion and early intervention programmes [19,21,22,24,25,26,27,28,29,30,31,32,34,35,36,37,41,43,45,46,47,48,49].

A total of 22 papers met or partially met at least one Priority of each Principle [20,21,22,23,25,26,27,28,29,30,31,32,33,34,35,36,37,38,40,41,44], with Principle 4 being the least met as discussed. The paper by Reeve et al. (2015) [35] was one of these. Their work evaluated the impact of changes to health performance indicators in a remote area of Australia after the introduction of a comprehensive primary health care service model. To enable changes to the health system, a formal partnership agreement between an acute health service provider, community health service providers, and the community-controlled health service was developed, a process which took one year [35]. This enabled shared funding and care planning/monitoring. More Indigenous-appropriate services were provided, and there was regular feedback from the local Indigenous community. Specific changes in response to this feedback and partnerships provided more person-centred care, including increased employment of Indigenous staff, cultural training for all staff, leading to “a better understanding of the importance of families and their guardianship roles”. More patient-support individuals stayed with patients as boarders; patient transport was increased to assist patients in attending appointments; and traditional healers were included as part of care, as were traditional ceremonies such as smoking of rooms after a death, and a less structured approach to appointments. The project was reviewed retrospectively over 6 years, and results included increased attendance at follow-up appointments and screening appointments, people attempting to quit smoking and reduce alcohol intake, a reduction in the number of deaths, and a decrease in the number of people requiring emergency evacuation from the hospital [35].

Gaps in alignment with the Framework Priorities were identified in areas of social and emotional wellbeing, mental health and suicide prevention, promoting healthy environments, sustainability and preparedness, and shared access to data and information. The understanding, awareness, and conceptualisation of Indigenous SEWB grew during the 12 years of UDRH publications [7]. This was evident in the fact that the four papers that aligned with Priority 6 were published in the later time period of papers included [22,35,38,45], when the awareness of SEWB was growing and was increasingly incorporated into Australian government policy [7].

As we were surprised by the lack of alignment with mental health research identified in this assessment, we further examined the original ARHEN database of publications in Indigenous health. Of the 493 articles in the database, there were 34 articles with topics regarding “mental health” or “suicide”, published between 2010 and 2021. Many of these articles were coded as “descriptive” or “measurement” rather than interventions, indicating that the gap in UDRH research was specifically in applying interventions that aim to improve mental health outcomes, rather than a lack of studies around mental health per se. However, it should be noted that community programmes and public health measures such as education campaigns to raise awareness of mental health risks, signs and support, and suicide prevention may be undertaken by UDRHs and partners, but they may not be reported as peer-reviewed publications [55]. Moreover, UDRHs may undertake interventions with health professionals and Indigenous community members without this being conceived or undertaken as research, but rather as evidence into practice.

Although only six articles showed evidence of alignment with Priority 12—shared access to data and information at a regional level [22,27,30,34,35,41]—this finding may not represent the actual practice of what occurred in the research and may also reflect word limit constraints within journals. However, there have been changes to practices since the promotion of Indigenous Data Sovereignty guidelines and the increasing capability of Indigenous researchers available to lead research [54]. Future UDRH publications should improve transparency in reporting on shared access to data and information. Promoting healthy environments, sustainability, and preparedness (Priority 7) were identified as gaps in UDRH intervention research, an unsurprising finding since this domain is outside of UDRHs’ core business of developing the rural health workforce, and an area which ARHEN and UDRHs have advocated needs reconsideration [56].

### Limitations

Our measurement of alignment of interventions to Principles and Priorities of the Framework can be viewed as a “report card”. We utilised papers already identified and coded through previous processes, so it is possible that not all relevant UDRH publications in this period were identified. Importantly, we note that any papers that met a larger number of the Priorities of the Framework do not mean the research is better or more valuable. Focused research in a narrow area may prove invaluable in a particular context and contribute to capacity building, policy, and funding. Mapping the alignment to the Framework was an exercise in taking a deeper look at how the UDRHs’ research incorporated existing and emerging understandings of Indigenous health policy into interventional projects with Indigenous people across 12 years. While the Framework used was published after the research assessed here was published, this analysis can inform approaches to interventions in Indigenous health in the future.

## 5. Conclusions

UDRHs have focused on building relationships with Indigenous Australians, linkages with regional health, and with primary healthcare services. This paper showed the consistent efforts of UDRHs to reduce the disparities in health between Indigenous and non-Indigenous Australians. Their footprint across Australia highlights the important role that rurally based academic centres can play in progressing national efforts to improve Indigenous health and wellbeing.

Overall, as previously identified, there has been a dearth of intervention studies and a need to move beyond descriptive studies, which, unfortunately, often document deficits. The alignment of studies with the contemporary and future-focused Indigenous Health Plan Vision can inform planning for future Indigenous health research across the national network and lead to meaningful improvements in Indigenous health.

Understanding of the Framework, additional efforts into recruitment and support of Indigenous staff within UDRHs, and local consultations when planning projects can help to ensure that Indigenous-led research informs research and project planning. The strong relationships with Indigenous stakeholders lead to the development of more culturally appropriate approaches to building capacity in the Indigenous health workforce [14]. UDRHs within the ARHEN are increasingly collaborating in research, and as well as individual Aboriginal staff employed in UDRHs, the Aboriginal and Torres Strait Islander Staff Network is an effective means for providing leadership. Through its focus on the Framework, this paper will inform UDRHs’ future efforts at a larger scale, significant intervention research led by rurally based Indigenous researchers across Australia.

## Figures and Tables

**Table 1:** Summary of the number of articles mapped to each Principle and Priority in the Framework (*n* = 33).

**Mapping**	**Principle 1** **Enablers for Change** ***n* (%)**			**Principle 2** **Focus on Prevention** ***n* (%)**				**Principle 3** **Improving the Health System** ***n* (%)**			**Principle 4** **Culturally Informed** **Evidence-Base *n* (%)**	
Priority	P1	P2	P3	P4	P5	P6	P7	P8	P9	P10	P11	P12
# Yes	22 (67)	13 (39)	19 (58)	21 (64)	15 (46)	4 (12)	0 (0)	33 (100)	8 (24)	3 (9)	19 (58)	6 (18)
# Partially	0	0	1	1	4	8	1	0	6	4	3	0
# No	11	20	13	11	14	21	32	0	19	26	11	27
# article coding against each Priority												
**Key—Health Plan Priorities**												
P1. Genuine shared decision making and partnerships P2. Aboriginal and Torres Strait Islander community-controlled comprehensive primary health care P3. Workforce P4. Health promotion P5. Early intervention P6. Social and emotional wellbeing and trauma-aware, healing-informed approaches						P7. Healthy environments, sustainability, and preparedness P8. Identify and eliminate racism P9. Access to person-centred and family-centred care P10. Mental health and suicide prevention P11. Culturally informed and evidence-based evaluation, research and practice P12. Shared access to data and information at a regional level						

## Data Availability

No new data were created or analyzed in this study. Data sharing is not applicable to this article.

## Supplementary Materials

The following supporting information can be downloaded at: https://www.mdpi.com/article/10.3390/ijerph23050600/s1. Table S1: Summary of all papers and mapping to the National Aboriginal and Torres Strait Islander Health Plan 2021–2031.

## Abbreviations

The following abbreviations are used in this manuscript:

ARHEN	Australian Rural Health and Education Network
UDRH	University Department of Rural Health
WACRH	Western Australian Centre for Rural Health
