# Supplementary material for: Looking Back to Move Forward: A Narrative Review of Indigenous Health Intervention Research by the University Departments of Rural Health Against a Contemporary National Framework
Source: Int J Environ Res Public Health. 2026 May 1;23(5):600. doi: 10.3390/ijerph23050600 (PMC13206609; doi:10.3390/ijerph23050600)
Supplement: Supplementary file 1 [file ijerph-23-00600-s001.zip › ijerph-4186269-supplementary.pdf]

International Journal of *Environmental Research and Public Health*

MDPI

Table S1: Summary of all papers and mapping to the National Aboriginal and Torres Strait Islander Health Plan 2021-2031 [2]

Article	Summary of the intervention	Sample size of intervention group (n=)	Evaluation based on the National Aboriginal and Torres Strait Islander Health Plan 2021-2031											
			Principle 1: Enablers for change			Principle 2: Focusing on prevention				Principle 3: Improving the health system			Principle 4: Culturally informed evidence base	
			P1	P2	P3	P4	P5	P6	P7	P8	P9	P10	P11	P12
Bailie et al. (2017) [20]	ABCD program to enable the use of CQI tools to improve best practices and quality of care.	175 Indigenous primary healthcare services	[green]	[green]	[yellow]	[yellow]	[yellow]	x	x	[green]	x	x	[green]	x
Bennett-Levy et al. (2017) [21]	Staff education on strategies related to overcoming the barriers and strengthening the enablers to the adoption of e-mental health services	26 participants 21 Indigenous and 5 non-Indigenous	[green]	x	[green]	[green]	x	x	x	[green]	x	[green]	[green]	x
Bennett-Levy et al. (2021) [22]	Digital social and emotional wellbeing: strategies that address cultural, social, and emotional needs of Indigenous populations.	5 Community partner organisations across 6 years	[green]	x	[green]	[green]	x	[green]	x	[green]	x	[green]	[green]	[green]
Biles et al. (2021) [23]	A mentoring program for Aboriginal and Torres Strait Islander nurses and midwives in a rural health district.	Unclear	[green]	x	[green]	x	x	[yellow]	x	[green]	x	x	[green]	x
Brimblecombe et al. (2017) [24]	Discounted healthy food and drinks in remote NT Indigenous	20 communities	[green]	x	x	[green]	[green]	x	x	[green]	x	x	x	x

Article	Summary of the intervention	Sample size of intervention group (n=)	Evaluation based on the National Aboriginal and Torres Strait Islander Health Plan 2021-2031											
			Principle 1: Enablers for change			Principle 2: Focusing on prevention				Principle 3: Improving the health system			Principle 4: Culturally informed evidence base	
			P1	P2	P3	P4	P5	P6	P7	P8	P9	P10	P11	P12
	communities, with and without consumer education.													
Cairns et al. (2022) [25]	Action research development of a co-designed community rehabilitation and disability support service in two remote QLD communities.	2 communities	[green]	x	x	[green]	x	x	x	[green]	[yellow]	x	[green]	x
Carey et al. (2016) [50]	A community based, culturally appropriate palliative care respite service. Patients were able to access the service when they needed to rather than attending a prescribed program and staff spent time understanding the individual requirements, including the cultural considerations of each patient.	20 participants	x	x	x	x	x	[yellow]	x	[green]	[green]	x	x	x
Chapple et al. (2016) [26]	Living Well Smoke Free (LWSF) training – a smoking cessation program adapted for Aboriginal and Torres Strait Islander people	10 participants	[green]	[green]	[green]	[green]	x	[yellow]	x	[green]	[yellow]	[yellow]	[green]	x
Durey et al. (2016) [27]	District Aboriginal Health Action Groups collaborated with health	60 participants	[green]	[green]	[green]	[green]	x	x	x	[green]	x	x	[green]	[green]

Article	Summary of the intervention	Sample size of intervention group (n=)	Evaluation based on the National Aboriginal and Torres Strait Islander Health Plan 2021-2031											
			Principle 1: Enablers for change			Principle 2: Focusing on prevention				Principle 3: Improving the health system			Principle 4: Culturally informed evidence base	
			P1	P2	P3	P4	P5	P6	P7	P8	P9	P10	P11	P12
	service providers to design culturally responsive care													
Fernando et al. (2021) [49]	Annual measurement of salivary flow and bacterial load with usual treatment compared with measurements, fissure sealant to appropriate teeth, swabbing with povidine iodine and application of fluoride varnish.	208 participants	X	X	X	X	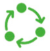	X	X	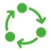	X	X	X	X
Guy et al. (2018) [42]	Point-of-care testing for chlamydia and gonorrhoea	12 health services	X	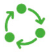	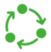	X	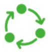	X	X	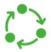	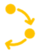	X	X	X
Haigh et al. (2016) [28]	Examination of the implementation and use of a DVD that was developed to educate Aboriginal people about bowel cancer and bowel cancer screening. Concluded that the content was acceptable but distribution was a barrier as it was delivered via post directly to participants rather than in person.	67 participants	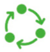	X	X	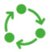	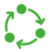	X	X	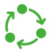	X	X	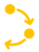	X

Article	Summary of the intervention	Sample size of intervention group (n=)	Evaluation based on the National Aboriginal and Torres Strait Islander Health Plan 2021-2031											
			Principle 1: Enablers for change			Principle 2: Focusing on prevention				Principle 3: Improving the health system			Principle 4: Culturally informed evidence base	
			P1	P2	P3	P4	P5	P6	P7	P8	P9	P10	P11	P12
Isaacs & Lampitt (2014) [29]	Koorie men's health day. Single pilot intervention where men had a complete medical exam, were screened for diabetes and had an assessment for psychological distress. All male health professionals. Driven by local Aboriginal community.	20 participants	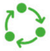	X	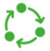	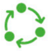	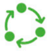	X	X	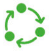	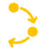	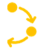	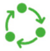	X
Khalil (2019) 30]	Medication safety education tailored for Aboriginal health practitioners	17 Aboriginal Health Practitioners	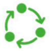	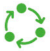	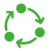	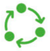	X	X	X	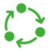	X	X	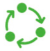	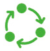
Kong et al. (2021) [31]	"Grinnin' Up Mums & Bubs": train Aboriginal Health Workers to promote oral health among Aboriginal and Torres Strait Islander pregnant women.	7 Aboriginal Health workers	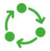	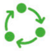	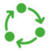	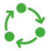	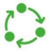	X	X	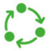	X	X	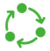	X
Lalloo et al. (2021) [47]	Part of the Fernando et al 2021. Dental intervention: placement of fissure sealants on suitable teeth, and application of povidone-iodine and fluoride varnish to the whole dentition, following completion of any	208 participants	X	X	X	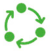	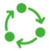	X	X	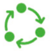	X	X	X	X

Article	Summary of the intervention	Sample size of intervention group (n=)	Evaluation based on the National Aboriginal and Torres Strait Islander Health Plan 2021-2031											
			Principle 1: Enablers for change			Principle 2: Focusing on prevention				Principle 3: Improving the health system			Principle 4: Culturally informed evidence base	
			P1	P2	P3	P4	P5	P6	P7	P8	P9	P10	P11	P12
	necessary restorative dental treatment. Standard diet and oral hygiene advice were provided.													
Lin et al. (2016) [45]	Education for General Practitioners for managing lower back pain (LBP): reducing unnecessary LBP radiological imaging referrals, enhancing psychosocial-oriented patient assessment, and increasing the provision of LBP self-management information to patients	6 GPs in one rural Australian Aboriginal Medical Service received the training. Measured change in pt care.	X	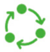	X	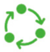	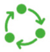	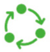	X	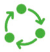	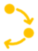	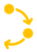	X	X
O'Connor et al. (2021) [44]	Employment of an Aboriginal Interpreter Coordinator, 'Working with Interpreters' training for healthcare providers, and championing of interpreter use by doctors.	One Aboriginal Interpreter coordinator<br>127 attendees to training sessions<br>3 clinical champions	X	X	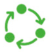	X	X	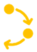	X	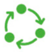	X	X	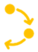	X

Article	Summary of the intervention	Sample size of intervention group (n=)	Evaluation based on the National Aboriginal and Torres Strait Islander Health Plan 2021-2031											
			Principle 1: Enablers for change			Principle 2: Focusing on prevention				Principle 3: Improving the health system			Principle 4: Culturally informed evidence base	
			P1	P2	P3	P4	P5	P6	P7	P8	P9	P10	P11	P12
		Over 12 months intervention												
Passey & Stirling (2018) [32]	Assessment of the feasibility and acceptability of a culturally tailored, intensive smoking cessation program for pregnant Aboriginal women that included contingency-based financial rewards.	22 participants	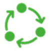	X	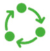	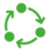	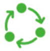	X	X	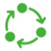	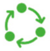	X	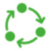	X
Prout et al. (2013) [33]	An experiential and hands-on approach to education in rural health settings: students were placed in real rural health contexts.	27 students	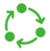	X	X	X	X	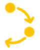	X	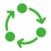	X	X	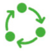	X
Rae et al. (2014) [34]	The Gomeroi gaaynggal program aimed at reducing renal disease in women and their children: an art group that meets weekly run by a local Indigenous artist and attended by health professionals, providing education to pregnant Indigenous	100 participants	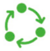	X	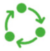	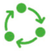	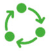	X	X	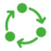	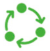	X	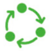	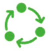

Article	Summary of the intervention	Sample size of intervention group (n=)	Evaluation based on the National Aboriginal and Torres Strait Islander Health Plan 2021-2031											
			Principle 1: Enablers for change			Principle 2: Focusing on prevention				Principle 3: Improving the health system			Principle 4: Culturally informed evidence base	
			P1	P2	P3	P4	P5	P6	P7	P8	P9	P10	P11	P12
	women about topics related to renal disease.													
Ralph et al. (2018) [46]	Stepped-wedge, randomized trial: a multicomponent intervention supporting activities to improve penicillin delivery, aligned with the chronic care model, with continuous quality-improvement feedback on adherence.	10 remote Australian Aboriginal clinics	X	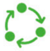	X	X	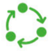	X	X	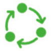	X	X	X	X
Read et al. (2018) [43]	Evaluation of the Ralph et al (2018) study to improve penicillin prophylaxis in Aboriginal individuals with rheumatic heart disease in 10 remote Aboriginal communities	10 communities<br>121 health-centre staff<br>22 informants<br>72 clients	X	X	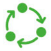	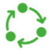	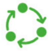	X	X	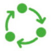	X	X	X	X
Reeve et al. (2014) [36]	Implementation of an ear health team in primary care to ensure high-quality referrals and a more patient-centred approach to continuity of care and follow up.	Control – 148 participants<br>Intervention – 710 participants	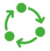	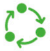	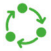	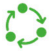	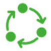	X	X	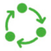	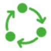	X	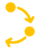	X
Reeve et al. (2015) [35]	Integration of policy implementation, health promotion, health	Health service data for all	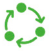	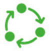	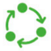	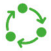	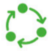	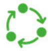	X	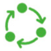	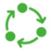	X	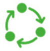	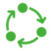

Article	Summary of the intervention	Sample size of intervention group (n=)	Evaluation based on the National Aboriginal and Torres Strait Islander Health Plan 2021-2031											
			Principle 1: Enablers for change			Principle 2: Focusing on prevention				Principle 3: Improving the health system			Principle 4: Culturally informed evidence base	
			P1	P2	P3	P4	P5	P6	P7	P8	P9	P10	P11	P12
	assessments and chronic disease management with an acute primary health care service through formal partnership between the hospital, community health service and community controlled health service.	residents in Fitzroy Valley over 6 years incl. reports for the three partner health service organisations delivering care in that area.												
Schoen et al. (2016) [19]	Education to enhance rural and remote practitioners' understanding of diabetic foot care.	246 healthcare professionals 2 rural and remote areas of WA	X	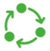	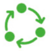	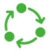	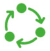	X	X	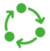	X	X	X	X
Shephard et al. (2016) [37]	Point-of-care pathology testing for diabetes management	Focus group x 1 Questionnaire sent to 470 point of care operators 104 completions	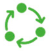	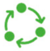	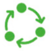	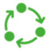	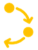	X	X	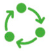	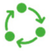	X	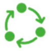	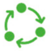

Article	Summary of the intervention	Sample size of intervention group (n=)	Evaluation based on the National Aboriginal and Torres Strait Islander Health Plan 2021-2031											
			Principle 1: Enablers for change			Principle 2: Focusing on prevention				Principle 3: Improving the health system			Principle 4: Culturally informed evidence base	
			P1	P2	P3	P4	P5	P6	P7	P8	P9	P10	P11	P12
Spaeth & Shephard (2016) [51]	Point-of-care testing to monitor the efficacy of warfarin therapy in Rheumatic Heart Disease management. This article reports on the operational and clinical benefits of INR testing in the program from 2008-2014	32 health services	X	X	X	X	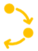	X	X	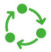	X	X	X	X
Thackrah & Thompson (2013a) [38]	University undergraduate tutorials about Aboriginal cultural safety and security for health science students: Aboriginal and Torres Strait Islander history, diversity, cultural protocols, social structures, patterns of communication, contemporary policies and their implications for health professionals.	15 students	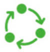	X	X	X	X	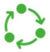	X	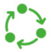	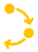	X	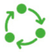	X
Thackrah & Thompson (2013b) [39]	University undergraduate tutorials about Aboriginal cultural safety and security for health science students: Aboriginal and Torres Strait Islander history, diversity, cultural protocols, social structures, patterns of	15 students	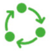	X	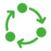	X	X	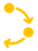	X	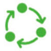	X	X	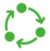	X

Article	Summary of the intervention	Sample size of intervention group (n=)	Evaluation based on the National Aboriginal and Torres Strait Islander Health Plan 2021-2031											
			Principle 1: Enablers for change			Principle 2: Focusing on prevention				Principle 3: Improving the health system			Principle 4: Culturally informed evidence base	
			P1	P2	P3	P4	P5	P6	P7	P8	P9	P10	P11	P12
	communication, contemporary policies and their implications for health professionals.													
Thackrah et al. (2015a) [48]	University undergraduate tutorials about Aboriginal cultural safety and security for health science students: Aboriginal and Torres Strait Islander history, diversity, cultural protocols, social structures, patterns of communication, contemporary policies and their implications for health professionals.	44 students	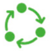	X	X	X	X	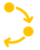	X	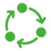	X	X	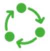	X
Thackrah et al. (2015b) [40]	A short rural clinical placement for midwifery students focused on antenatal care, sexual health, breast screening and promotion of healthy behaviours. Semi-structured interviews	7 students	X	X	X	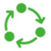	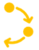	X	X	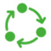	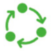	X	X	X
Tsey et al. (2014) [41]	An educational module about Aboriginal and Torres Strait Islander	9 trainers<br>16 learners	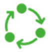	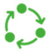	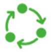	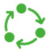	X	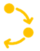	X	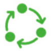	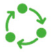	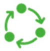	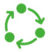	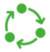

Article	Summary of the intervention	Sample size of intervention group (n=)	Evaluation based on the National Aboriginal and Torres Strait Islander Health Plan 2021-2031											
			Principle 1: Enablers for change			Principle 2: Focusing on prevention				Principle 3: Improving the health system			Principle 4: Culturally informed evidence base	
			P1	P2	P3	P4	P5	P6	P7	P8	P9	P10	P11	P12
	male health: 15 units across a range of health areas													

Key			
Yes	Partially	Not evident	Health Plan Priorities
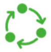	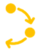	X	P1. Genuine shared decision making and partnerships<br>P2. Aboriginal and Torres Strait Islander community controlled comprehensive primary health care<br>P3. Workforce<br>P4. Health promotion<br>P5. Early intervention<br>P6. Social and emotional wellbeing and trauma-aware, healing-informed approaches<br>P7. Healthy environments, sustainability, and preparedness<br>P8. Identify and eliminate racism<br>P9. Access to person-centred and family centred care<br>P10. Mental health and suicide prevention<br>P11. Culturally informed and evidence-based evaluation, research and practice<br>P12. Shared access to data and information at a regional level
